# Preclinical Pharmacology of BA-TPQ, a Novel Synthetic Iminoquinone Anticancer Agent

**DOI:** 10.3390/md8072129

**Published:** 2010-07-13

**Authors:** Scharri J. Ezell, Haibo Li, Hongxia Xu, Xiangrong Zhang, Evrim Gurpinar, Xu Zhang, Elizabeth R. Rayburn, Charnell I. Sommers, Xinyi Yang, Sadanandan E. Velu, Wei Wang, Ruiwen Zhang

**Affiliations:** 1 Division of Clinical Pharmacology, Department of Pharmacology and Toxicology, University of Alabama at Birmingham, Birmingham, AL 35294, USA; E-Mails: sjezell@uab.edu (S.J.E.); haibo.li@ccc.uab.edu (H.L.); hxxu@uab.edu (H.X.); xiangrong.zhang@ccc.uab.edu (X.Z.); egurpinar@uab.edu (E.G.); xu.zhang@ccc.uab.edu (X.Z.); erayburn@uab.edu (E.R.R.); charnell.sommers@ccc.uab.edu (C.I.S.); xinyiy@uab.edu (X.Y.); wei.wang@ccc.uab.edu (W.W.); 2 Department of Chemistry, University of Alabama at Birmingham, Birmingham, AL 35294, USA; E-Mail: svelu@uab.edu; 3 Comprehensive Cancer Center, University of Alabama at Birmingham, Birmingham, Al 35294, USA; 4 College of Preventive Medicine, The Third Military Medical University, Chongqing 400038, China

**Keywords:** marine alkaloid, pharmacokinetics, protein binding, chemotherapy

## Abstract

Marine natural products and their synthetic derivatives represent a major source of novel candidate anti-cancer compounds. We have recently tested the anti-cancer activity of more than forty novel compounds based on an iminoquinone makaluvamine scaffold, and have found that many of the compounds exert potent cytotoxic activity against human cancer cell lines. One of the most potent compounds, BA-TPQ [(11,12),7-(benzylamino)-1,3,4,8-tetrahydropyrrolo[4,3,2-de]quinolin-8(1H)-one], was active against a variety of human cancer cell lines, and inhibited the growth of breast and prostate xenograft tumors in mice. However, there was some toxicity noted in the mice following administration of the compound. In order to further the development of BA-TPQ, and in a search for potential sites of accumulation that might underlie the observed toxicity of the compound, we accomplished preclinical pharmacological studies of the compound. We herein report the *in vitro* and *in vivo* pharmacological properties of BA-TPQ, including its stability in plasma, plasma protein binding, metabolism by S9 enzymes, and plasma and tissue distribution. We believe these studies will be useful for further investigations, and may be useful for other investigators examining the use of similar compounds for cancer therapy.

## 1. Introduction

Chemotherapy is frequently used to treat cancer, particularly advanced and metastatic cancers. Unfortunately, chemotherapeutic agents also often exert their toxic effects in rapidly-dividing non-malignant cells, including cells of the bone marrow and digestive tract, and hair follicles. Moreover, acquired and intrinsic resistance to chemotherapeutic agents is a major obstacle to successful cancer treatment. Therefore, there is an urgent need for the development of novel, effective therapeutic agents with novel or multiple mechanisms of action, and limited host toxicity.

Natural products have proven to be an abundant source of anti-cancer compounds. Several therapeutic agents currently used in the clinic, such as paclitaxel, doxorubicin, and vincristine, are derivatives of natural products. Among the many natural compounds being evaluated for their anticancer activity, we and others have observed that compounds of the makaluvamine sub-class have especially potent activity [1–4]. Previous studies have shown that therapeutic agents belonging to this class exert their anti-cancer activity by inhibiting the enzymatic activity of topoisomerase II [4–6], a protein that facilitates the uncoiling of DNA during replication. Topoisomerases (type I and II) have proven to be valid targets for therapy, and various clinically-used chemotherapeutic agents, including irinotecan (Top I), topotecan (Top 1), and etoposide (Top II), act by inhibiting these enzymes [7,8].

**Figure 1:**
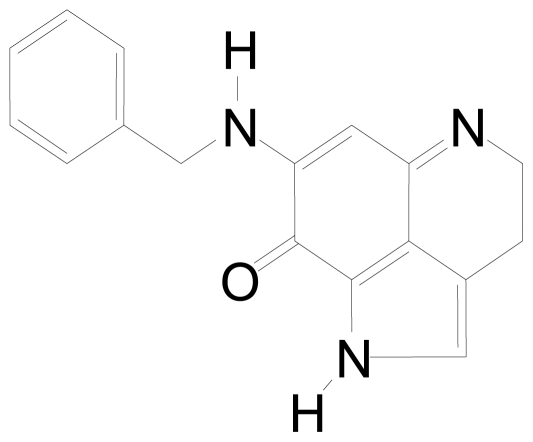
Structure of BA-TPQ.

We recently developed a novel method for the total chemical synthesis of makaluvamine analogs [9], and a series of these analogs were screened using the National Cancer Institute panel of 60 human cancer cell lines [10]. Although a number of the makaluvamine analogs exhibited anti-cancer activity, [11,12], 7-(benzylamino)-1,3,4,8-tetrahydropyrrolo[4,3,2-de]quinolin-8(1H)-one (BA-TPQ, Figure 1) was the most potent inducer of apoptosis and cell cycle arrest. Moreover, BA-TPQ was found to inhibit *in vitro* growth of prostate and breast cancer cell lines with varying p53 status and to inhibit tumor growth in mouse xenograft models of these cancers [11,13].

Of interest, unlike other makaluvamine compounds (which exert their activity via inhibition of topoisomerase II), BA-TPQ appears to exert its anti-cancer effects through multiple mechanisms of action, including inhibition of the MDM2 oncoprotein, induction of the DNA damage response, and induction of ER (endoplasmic reticulum) stress [11,13]. This novel compound is currently undergoing preclinical development as a potential anti-cancer agent. Herein, we report the results of preclinical investigations evaluating the pharmacological properties of BA-TPQ in *in vitro* and *in vivo* systems. We believe that these results will support the future preclinical and clinical evaluation of this synthetic iminoquinone as a novel anti-cancer therapeutic.

## 2. Results and Discussion

### 2.1. In vitro stability of BA-TPQ in mouse plasma

We examined the stability of BA-TPQ at 37 °C, 4 °C, and −80 °C in mouse plasma. Under all of these conditions, BA-TPQ was very stable at both the low (0.5 μM) and high (5.0 μM) concentrations over the duration of time examined (Table 1).

### 2.2. In vitro protein binding of BA-TPQ

Considering the high *in vitro* stability of BA-TPQ, we believed that the compound might bind extensively to plasma proteins. To examine this possibility, pooled murine plasma samples were examined for their capacity to bind the iminoquinone at concentrations of 0.5 μM and 5 μM. BA-TPQ was considerably bound to proteins in mouse plasma, with 63% ± 37.9%; and 74% ± 2.3%; of the compound being bound to plasma proteins at concentrations of 0.5 μM and 5.0 μM, respectively.

### 2.3. S9 metabolism

To accomplish a preliminary study of the metabolism of BA-TPQ, the compound was incubated with S9 fractions containing Phase I and Phase II metabolic enzymes and their co-factors for 15, 30, 45, or 60 min. These studies indicated that BA-TPQ undergoes considerable metabolism in the presence of both Phase I and Phase II enzymatic components, with approximately 16% of the compound being metabolized in each set of reagents (Phase I: 16.8% ± 8.1%; Phase II: 16.4% ± 5.4%) during the 60 min incubation (data not shown).

### 2.4. Pharmacokinetics of BA-TPQ in mice

**Figure 2:**
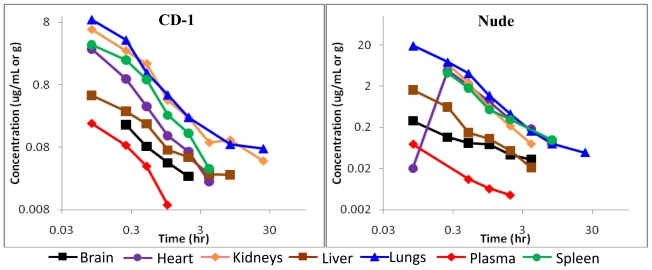
Concentration-time curves in mice dosed intravenously (5 mg/kg) with BA-TPQ. The x- and y-axes are plotted on a logarithmic scale. (Left panel: CD-1 mice; Right panel: Nude mice).

**Figure 3:**
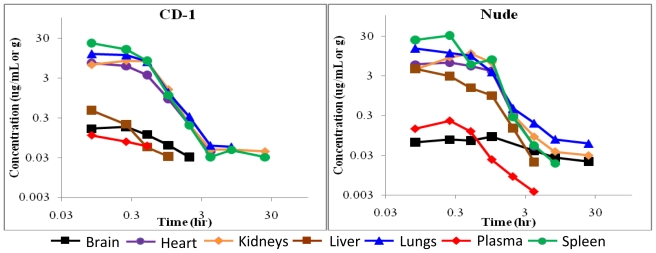
Concentration-time curves for BA-TPQ administered by intraperitoneal injection (10 mg/kg). The x- and y-axes are plotted on a logarithmic scale. (Left panel: CD-1 mice; Right panel: Nude mice).

To assess the disposition of BA-TPQ *in vivo*, pharmacokinetic studies in CD-1 and nude mice were conducted. The results are presented in Figures 2 and 3. Pharmacokinetic modeling revealed that a two compartment model (WinNonlin 6.0 Model 7) best fit the concentration-time profile for the intravenous (IV) administration of BA-TPQ, while a non-compartmental model was used to analyze the data obtained following intraperitoneal (IP) administration of the compound. The pharmacokinetic parameters of BA-TPQ in CD-1 and nude mice are presented in Tables 2 and 3, respectively.

Following a single bolus intravenous dose of BA-TPQ (5 mg/kg), there were relatively high concentrations of the compound in the plasma for the first 30 minutes (up to 0.18 μg/mL and 0.29 μg/mL; Figure 2a and 2b, for CD-1 and nude mice, respectively), with a rapid decline thereafter, as indicated by a half-life of 0.14 h and 0.17 h, and a clearance rate of 4.92 × 10^5^ mL/h/kg and 9.75 × 10^5^ mL/h/kg in CD-1 and nude mice, respectively (Tables 2 and 3). The iminoquinone was no longer detectable in plasma after 2 h post-injection in either strain of mice. Conversely, the compound was still detectable in the lungs of both CD-1 and nude mice at 24 h after intravenous administration of BA-TPQ. The amount of BA-TPQ excreted in the urine of both strains of mice was less than 0.1% of the administered dose, and the amount excreted in feces was less than 1% of the administered dose (data not shown).

BA-TPQ plasma concentrations following intraperitoneal dosing (10 mg/kg) were greatest at 15 min for CD-1 mice (0.18 μg/mL) and 1 h for nude mice (0.09 μg/mL), indicating that BA-TPQ is rapidly absorbed from the peritoneal cavity (Figure 3a and 3b). In contrast to intravenous dosing, intraperitoneal administration generally led to higher AUC values (a measure of the total exposure of the plasma or a particular tissue to the drug) in plasma and tissues in both strains, as indicated in Tables 2 and 3. Similar to IV dosing, low concentrations of BA-TPQ were present in the urine collected for the first 24 h after dosing, with less than 0.02% of the administered dose being excreted in the urine, and less than 1% being excreted in the feces following IP administration of a 10 mg/kg dose of the compound (data not shown). The S9 data indicate that the compound is fairly extensively metabolized by microsomal enzymes, and the low excretion of the intact compound confirms this.

The accumulation of BA-TPQ in the liver of nude mice following intraperitoneal injection was much higher (~18-fold) than for CD-1 mice, and the AUC in the brain of the nude mice was also increased (~5-fold) compared to CD-1 mice following intraperitoneal injection. Similarly, the exposure of the heart of nude mice to BA-TPQ following intravenous injection was approximately 6-fold that of CD-1 mice (as measured by AUC; Tables 2 and 3). No strain differences were observed in the plasma pharmacokinetic profile of BA-TPQ following either IP or IV administration. In general, the spleen, lungs, kidneys and heart had relatively high concentrations of BA-TPQ compared to the plasma, brain and liver, regardless of the route of administration or the strain of mice.

In fact, while there were a few differences between parameters for a few tissues, our data indicated that overall there were not any major differences in the distribution of BA-TPQ between the two strains of mice following IV or IP administration. The intraperitoneal bioavailability of BA-TPQ in CD-1 and nude mice was 74% and 100%, respectively. However, the concentrations detected in plasma were relatively low, and with some variability, so we believe that the true intraperitoneal bioavailability of BA-TPQ is likely closer to that in the CD-1 mice (~70%).

### 2.5. Discussion

Recent studies have demonstrated the ability of BA-TPQ to inhibit the survival of breast, colon, prostate, and lung cancer cells *in vitro* [9–12], and decrease tumor growth *in vivo* [11,13]. Although a number of makaluvamine analogs have been reported to inhibit topoisomerase activity [9], recent data indicates that BA-TPQ acts through a variety of mechanisms, leading to the induction of apoptosis and inhibition of cell cycle progression [11–13]. Unfortunately, while the compounds were highly effective, their use in mice appears to produce systemic toxicity (as indicated by weight loss), especially at the higher dose of 10 mg/kg when it was given 3 days/week for several weeks. While the lower doses (1 mg/kg and 5 mg/kg) were effective, we would like to be able to increase the therapeutic window of the compound by changing the dosing frequency, the formulation, or other factors related to the distribution of the compound. However, we did not previously have any knowledge about how the compound is distributed, and did not observe any gross organ abnormalities that indicated a particular target tissue. Our studies reported herein represent an effort to describe the clinical pharmacology of BA-TPQ, a novel synthetic marine natural product that may be valuable in cancer therapy.

Preclinical pharmacokinetic studies aid in the determination and optimization of various therapy-related parameters, including the dosing, frequency of administration, formulation, and potential sites of toxicity of the agent. Such information is essential to the development of a compound toward Phase I clinical trials. Continuing our pre-clinical work with the compound, we have evaluated the pharmacokinetic profile of BA-TPQ in the plasma and tissues of CD-1 and nude mice.

As a preliminary assessment of potential toxic side effects, including cardio- and hepato-toxicity [14,15], we have evaluated the distribution and accumulation of BA-TPQ in plasma and various tissues in order to determine whether specific organs are being targeted by this compound. Although most pharmacokinetic studies report only the plasma pharmacokinetics of a compound, we believe that it is critical to assess the distribution of candidate anti-cancer therapeutics in various tissues, both to determine potential sites of toxicity and to assess the overall uptake and bioavailability of the compound following administration by different routes or in different formulations.

The pharmacokinetic profile for tissues obtained in our study suggests that there is high drug accumulation in the lungs, kidneys, and spleen of the mice, and that it reaches low concentrations in the brain. Furthermore, pharmacokinetic modeling confirmed that these tissues exhibited the highest drug exposure (AUC), longest half-life, and lowest clearance values, indicating that these organs may be potential sites of BA-TPQ toxicity (perhaps explaining the toxicity observed during the initial *in vivo* studies in tumor-bearing mice), or that they may be ideal tumor sites to target using the drug. With regard to toxicity, it appears that BA-TPQ may be less toxic than other natural products from marine sponges, because it does not cause death in mice given repeated doses of 10 mg/kg (three times per week for up to six weeks [13]) [16,17]. In fact, it appears to have toxicity similar to the marine natural products used in the clinic [14,15], suggesting that the compound may be sufficiently tolerable even using the current formulation. In addition, while the AUC and clearance for these particular tissues may be slightly sub-optimal with regard to toxicity, they are not unusual for such a compound. Moreover, the data obtained during our study were fairly consistent with other clinically-used compounds, and was in the expected range based on published studies of intravenous infusion of Yondelis (trabectedin, ET-743), a marine alkaloid currently in phase II/III clinical trials [18,19].

We did not examine the distribution of the compound in the mammary tissue or prostate in the mice, as there are major differences in these tissues between humans and mice (particularly with regard to the prostate architecture). However, we did note high levels of the compounds in the lungs, suggesting that the compound may be even more effective for lung cancer than for breast and prostate cancer. Of interest, our pharmacokinetic data also showed that the compound has an AUC in the brain comparable to that in the plasma, indicating that the drug can cross the blood-brain barrier and may be effective for neoplasms in the brain.

While assessing the toxicity and ADME (absorption, distribution, metabolism, excretion) of a compound in a single mouse strain may be sufficient, we wanted to determine whether there were any differences in the disposition of the compound in different strains of mice. In addition to inter-species differences in ADME and toxicity (ADMET), there can also be strain-specific differences [20–22]. The athymic nude and C57Bl/6 strains of mice are the most frequently employed strains for studying the anti-cancer activity of a candidate therapeutic compound [23,24]. In contrast, CD-1 mice are typically chosen for evaluations of the safety and toxicological profiling of candidate anti-cancer agents because of their robust health, availability in large numbers, and their low cost (compared to nude and C57Bl/6 mice). Given the possible strain-dependent differences in ADMET, it is unclear whether the toxicity and pharmacology of the compound would be the same in mice used for efficacy studies and those conventionally used for toxicology and pharmacology studies. In our present study, we did not observe any major differences between strains, demonstrating that CD-1 mice can be used to predict the pharmacokinetics in nude mice.

Our study also investigated the pharmacokinetic profile of BA-TPQ following both intravenous (5 mg/kg) and intraperitoneal (10 mg/kg) administration. Although intraperitoneal administration is rarely used for clinical chemotherapy, it is occasionally used when the cancer site is localized within the intraperitoneal cavity (*i.e.*, ovarian cancer). In addition, IP administration of experimental therapeutics to animal models is the easiest way to assess the potential anti-cancer activity of the compound. However, because intravenous dosing is more clinically-relevant, and because the calculation of various values (e.g., bioavailability) is based upon the results of IV dosing, we also examined the pharmacokinetics of the compound following intravenous administration. Our data indicate that BA-TPQ possesses a short plasma half-life, regardless of the route of administration and the strain of mouse. We also observed that the bioavailability following IP injection was relatively high (>70%). This suggests that, in order to achieve better efficacy in models of cancer (compared to our findings in a previous study [13]), a new formulation or better delivery system may be useful.

Extensive binding of a candidate compound to plasma proteins can greatly impair its biodistribution and, consequently, its therapeutic efficacy. Although the unbound fraction of a particular drug can traverse the cell membrane more easily, and thus more easily exert its pharmacological effect, decreased binding to plasma proteins also renders the compound more vulnerable to metabolic enzymes. Therefore, compounds that are highly bound to proteins display longer half-lives, durations of action, and higher volumes of distribution. In the present study, BA-TPQ exhibited considerable protein binding, with approximately 70% of the compound bound to plasma proteins. This binding may have limited the *in vitro* degradation of the compound by the S9 enzymes. It is possible that the protein binding profile of BA-TPQ will be altered if it is used in combination with other therapeutic agents, depending on the affinity of these agents for plasma proteins. Such a possibility will need to be examined if the compound is tested in the clinic in order to rule out potential adverse interactions with other therapeutic agents or supplements.

During the evaluation of the metabolism of BA-TPQ by murine S9 microsomal fractions, we observed modest degradation of the compound. The subcellular (cytosolic and microsomal) liver fraction contains Phase I and Phase II metabolic enzymes, including cytochromes p450, flavin monooxygenases, and UDP glucuronyl transferases [25], all of which are replicated in the *in vitro* experiment. Our data suggest that BA-TPQ is metabolized by both Phase I and II metabolic enzymes, and that this process may be impeded by the considerable protein binding of the compound. No specific peaks that seemed to correspond to unique metabolites were identified using the current HPLC method.

## 3. Experimental

### 3.1. Test compound, chemicals and reagents

BA-TPQ was kindly provided by Dr. Sadanandan Velu (University of Alabama at Birmingham). Acetonitrile and methanol were purchased from Fisher Scientific (Atlanta, GA). All other chemicals were purchased from Sigma Chemical Company (St. Louis, MO). Heparinized samples of non-Swiss albino mouse plasma were obtained from Lampire Biological Laboratories (Pipersville, PA). Hepatic S9 fractions (20 mg/mL) from male ICR/CD-1 mice were purchased from Celsis In Vitro Technologies (Chicago, IL). All chemicals and solvents used for sample preparation and high-performance liquid chromatography (HPLC) analysis were of analytical grade.

### 3.2. Instrumentation and chromatographic conditions

A high performance liquid chromatography (HPLC) analytical method for BA-TPQ quantitation was previously developed and validated. The HPLC system consisted of an Agilent 1120 Compact HPLC system equipped with a UV detector. Separation and quantification of the test compound was achieved using a Zorbax SB-C18 (5 μm, 4.6 × 150 mm) analytical column (Agilent Technologies, CA, USA) protected by a SB-C18 guard column (Agilent Technologies, CA, USA). The instrument was interfaced with the EZ-Chrom Elite Compact software provided by Agilent Technologies. The mobile phase was composed of 28.0% acetonitrile containing 0.03% formic acid. The column temperature was set at 35 °C under a gradient flow rate which was maintained at 0.5 mL/min for 7 min, increased to 1.2 mL/min at 8 min, and finally decreased to 0.5 mL/min at 11 min for the remainder of the 16 min run. The detection wavelength was set at 346 nm and 50 μL of each sample was injected into the HPLC system.

### 3.3. Stability in mouse plasma

The stability of BA-TPQ was evaluated at two concentrations (0.5 and 5.0 μM). The compound was incubated under the following conditions: 37 °C for 0, 1, 2, 4, and 8 h; 4 °C for 0, 1, 4, 8, and 24 h; and −80 °C for 0, 1, 2, and 4 weeks. At the designated time points, the samples were extracted and analyzed using the established HPLC analytical method. The stability of the iminoquinone was assessed by comparing the initial concentration (time 0) with the final concentration following incubation of the compound at the noted temperatures, and expressing this difference as a percentage of the initial concentration.

### 3.4. Binding to mouse plasma proteins

The extent of binding to mouse plasma proteins by BA-TPQ was determined using a micro-ultrafiltration system as previously described [26]. BA-TPQ was dissolved in methanol and added to pooled mouse plasma to yield final concentrations of 0.5 μM and 5.0 μM. Control solutions were prepared in methanol to account for non-specific binding of BA-TPQ to the filter membranes. Duplicate preparations of each concentration were incubated at 37 °C for 1 h, before being placed into the sample reservoirs of Amicon Centrifree® ultrafiltration tubes (30 kDa exclusion; Millipore Corporation, Bedford, MA, USA). The filter systems were then centrifuged at 3000 *g* (37 °C) for 30 min or until no solution remained in the reservoir. Aliquots were taken from each filtrate and analyzed using the validated HPLC method. The concentrations of the unfiltered plasma and control preparations were also determined using the same protocol and expressed as the “total drug” (T) present, while the amounts of BA-TPQ present in the filtrates were designated as “free drug” (F). The percentage of BA-TPQ bound to plasma proteins was calculated as follows:

(1) Percent Bound=[(T-F)/T]×100

### 3.5. S9 metabolism

A preliminary study of the metabolism of BA-TPQ was done using murine hepatic S9 fractions containing Phase I and II metabolic enzymes. The reaction mixture contained 10 μM BA-TPQ (in methanol), 1 mg/mL S9 and 100 mM Tris buffer (pH 7.4). (Experimental controls did not contain the hepatic S9 fractions.) Metabolic reactions were initiated by adding NADPH-regenerating systems to the reaction mixtures, and samples were incubated in a water bath at 37 °C. Duplicate aliquots of the mixtures were taken at 0, 15, 30, 45, and 60 min, and the samples were processed and analyzed. The percent of BA-TPQ that was metabolized by Phase I and Phase II enzymes was calculated by comparing the initial concentration of the iminoquinone to the measured concentration following incubation.

### 3.6. Pharmacokinetic studies

#### 3.6.1. Animals

Female ICR/CD-1 (18–20 g) mice were purchased from Harlan Laboratories (Indianapolis, IN), and female nude mice (nu/nu; 4–6 weeks) were purchased from Frederick Cancer Research and Development Center (Frederick, MD). All animal use and care procedures were approved by the Institutional Animal Care and Use Committee (IACUC) of the University of Alabama at Birmingham. All animals were fed with commercial diet and water *ad libitum* and were on an alternating 12 h light/dark cycle.

#### 3.6.2. Animal dosing and sampling

CD-1 and nude mice were randomly divided into groups of three, and were dosed either intravenously (5 mg/kg) or intraperitoneally (10 mg/kg) with BA-TPQ in PEG 400:ethanol:0.9% saline (57.1%:14.3%:28.6%). Before dosing, and at 5, 15, 30, 60 min, and 2, 4, 8, and 24 h after dosing, groups of animals (three/time point) were anesthetized, and blood was collected from the retro-orbital sinus into heparinized tubes. Plasma was obtained by centrifuging the blood samples at 14,000 rpm for 15 min. BA-TPQ was then extracted from plasma and stored at −80 °C. The brain, heart, kidneys, liver, lungs, and spleen were also collected at the same time points during necropsy, blotted on Whatman No. 1 filter paper, trimmed of extraneous fat and connective tissue, weighed, and homogenized in phosphate-buffered saline. The resulting homogenates were stored at −80 °C until further processing and analysis. Urine and feces were collected for 24 hr from mice housed in metabolic cages. After collection, the cages were also washed twice with 200 μL PBS, and the washes were collected and analyzed separately. The total urine was determined as the amount from the urine plus washes 1 and 2. Feces were homogenized in PBS, then extracted using the same procedure used for tissues.

#### 3.6.3. Sample preparation

Six hundred microliters of acetonitrile was added to 200 μL of plasma, urine, cage washes (first and second), or tissue/feces homogenate in an Eppendorf tube to precipitate proteins. After centrifugation at 14,000 rpm for 10 min, the supernatant was transferred to a glass tube and evaporated to dryness using a TurboVap® LV Concentration Workstation (Caliper Lifesciences, Hopkinton, MA). The residue was reconstituted with 500 μL ethyl acetate, sonicated for 10 min, and vortexed for 10 sec. Samples were then centrifuged again and the resulting supernatant was transferred to a new glass tube and evaporated to dryness. Finally, samples were reconstituted in 100 μL mobile phase, and 50 μL was injected into the HPLC system for analysis.

#### 3.6.4. Pharmacokinetic analysis

The averages of the BA-TPQ concentration in plasma and tissues following intravenous and intraperitoneal administration to three animals per time point were used for pharmacokinetic analyses using a two-compartmental model and a non-compartment model, respectively. The following pharmacokinetic parameters were estimated using Phoenix WinNonlin 6.0 (Mountain View, CA): area under the concentration-time curve (AUC, h·μg/mL), half-life (T_½_, h), maximum concentration (C_max_, μg/mL), time of the maximum concentration (T_max_, h), and clearance (CL, mL/h/kg), as applicable. Bioavailability (X) was calculated as X= [AUC_ip_ × dose_iv_]/[AUC_iv_ × dose_ip_].

## 4. Conclusions

In conclusion, the present study has uncovered the preclinical *in vitro* and *in vivo* pharmacological properties of a novel analog of a natural product derived from a marine sponge. Further studies employing mass spectrometry would be informative in order to identify whether there are specific metabolites of the compound that are responsible for its activity and/or toxicity. Moreover, since the activity of metabolic enzymes may vary between species, the metabolism of BA-TPQ by S9 fractions from other species, including humans, should also be investigated during the further pre-clinical evaluation of the compound. However, collectively, the data presented herein provide the basis for future evaluations of the pharmacology and toxicology of BA-TPQ as it continues to be developed as a novel anti-cancer therapeutic agent.

## Figures and Tables

**Table 1:** Stability of BA-TPQ stored in mouse plasma at different temperatures.

Temperature	Duration of Time	0.5 μM		5.0 μM	
		Amount Remaining (%)	RSD (%)	Amount Remaining (%)	RSD (%)
37 °C	8 hr	92	0.22	114	0.60
4 °C	24 hr	94	12	117	7
−80 °C	4 weeks	95	0.36	100	0.13

RSD (%): Relative standard deviation.

**Table 2:** Pharmacokinetic parameters for BA-TPQ following intravenous or intraperitoneal injection in CD-1 mice.

Tissue	Route	Dose (mg/kg)	R^2^*	C_max_ (μg/mL)	T_max_ (h)	T_1/2_ (h)	AUC (h·μg/mL)	Cl (mL/h/kg)
**Plasma**	IV	5	1.00	0.36	-	0.14	0.07	4.92 × 10^5^
	IP	10	0.95	0.11	0.08	0.86	0.11	3.45 × 10^5^
**Heart**	IV	5	1.00	5.34	-	0.13	1.02	0.34 × 10^5^
	IP	10	0.97	7.24	0.08	0.71	4.66	0.08 × 10^5^
**Lungs**	IV	5	1.00	13.05	-	0.26	4.98	0.07 × 10^5^
	IP	10	0.92	12.38	0.08	0.50	9.15	0.04 × 10^5^
**Liver**	IV	5	1.00	0.69	-	0.65	0.65	0.54 × 10^5^
	IP	10	0.88	0.47	0.08	0.24	0.15	2.46 × 10^5^
**Kidneys**	IV	5	1.00	9.68	-	0.20	2.83	0.12 × 10^5^
	IP	10	0.92	8.36	0.25	0.48	8.06	0.04 × 10^5^
**Spleen**	IV	5	1.00	4.59	-	0.27	1.80	0.19 × 10^5^
	IP	10	0.92	23.00	0.08	0.41	11.26	0.03 × 10^5^
**Brain**	IV	5	1.00	0.75	-	0.21	0.22	1.58 × 10^5^
	IP	10	0.97	0.18	0.25	0.83	0.19	1.75 × 10^5^

C_max_: maximum concentration of the compound observed; T_max_: time when the maximum concentration was observed; T_1/2_: half-life of the compound; AUC: area under the concentration-time curve; Cl: clearance

* R^2^: correlation coefficient of the data for the chosen WinNonlin pharmacokinetic model.

**Table 3:** Pharmacokinetic parameters for BA-TPQ following intravenous or intraperitoneal administration in nude mice.

Tissue	Route	Dose (mg/kg)	R^2^	C_max_ (μg/mL)	T_max_ (h)	T_1/2_ (h)	AUC (h·μg/mL)	Cl (mL/h/kg)
**Plasma**	IV	5	1.00	0.15	-	0.17	0.04	9.75 × 10^5^
	IP	10	0.95	0.22	0.25	1.17	0.15	2.35 × 10^5^
**Heart**	IV	5	1.00	48.80	-	0.09	6.37	0.05 × 10^5^
	IP	10	0.95	6.38	0.25	0.50	7.63	0.05 × 10^5^
**Lungs**	IV	5	0.95	18.38	-	0.35	9.18	0.04 × 10^5^
	IP	10	0.92	14.75	0.08	0.59	13.16	0.03 × 10^5^
**Liver**	IV	5	1.00	2.71	-	0.18	0.71	0.49 × 10^5^
	IP	10	0.98	4.56	0.08	0.53	2.76	0.13 × 10^5^
**Kidneys**	IV	5	1.00	20.76	-	0.18	5.48	0.06 × 10^5^
	IP	10	0.89	10.84	0.5	0.49	12.62	0.03 × 10^5^
**Spleen**	IV	5	1.00	10.99	-	0.31	4.98	0.07 × 10^5^
	IP	10	0.90	31.22	0.25	1.65	18.29	0.02 × 10^5^
**Brain**	IV	5	1.00	0.62	-	0.38	0.34	1.04 × 10^5^
	IP	10	0.92	0.09	1.00	4.06	0.77	0.39 × 10^5^

## Abbreviations

BA-TPQ: [(11,12),7-(benzylamino)-1,3,4,8-tetrahydropyrrolo[4,3,2-de]quinolin-8(1H)-one]
C_max_: Maximum concentration
T_max_: Time at maximum concentration
T_1/2_: Half-life
AUC: Area under the concentration-time curve
Cl: Clearance
ADME(T): Absorption distribution metabolism excretion (toxicity)

## References

[1] Newman DJ Cragg GM Marine natural products and related compounds in clinical and advanced preclinical trials J. Nat. Prod 2004 8 1216 1238 15332835 10.1021/np040031y

[2] Guzmán E Johnson J Carrier M Meyer C Pitts T Gunasekera S Wright A Selective cytotoxic activity of the marine-derived batzelline compounds against pancreatic cancer cell lines Anticancer Drugs 2009 20 149 155 19209032 10.1097/CAD.0b013e32831fa39e PMC3031457

[3] Hoang H LaBarbera D Mohammed K Ireland C Skibo E Synthesis and biological evaluation of imidazoquinoxalinones, imidazole analogues of pyrroloiminoquinone marine natural products J. Med. Chem 2007 50 4561 4571 17705462 10.1021/jm0700870

[4] Byler K Wang C Setzer W Quinoline alkaloids as intercalative topoisomerase inhibitors J. Mol. Model 2009 15 1417 1426 19424733 10.1007/s00894-009-0501-6

[5] Delfourne E Analogues of marine pyrroloiminoquinone alkaloids: synthesis and antitumor properties Anticancer Agents Med. Chem 2008 8 910 916 19075573 10.2174/187152008786847710

[6] Marshall K Andjelic C Tasdemir D Concepción G Ireland C Barrows L Deoxyamphimedine, a pyridoacridine alkaloid, damages DNA via the production of reactive oxygen species Mar. Drugs 2009 7 196 209 19597581 10.3390/md7020196 PMC2707043

[7] Barrows LR Radisky DC Copp BR Swaffar DS Kramer RA Warters RL Ireland CM Makaluvamines, marine natural products, are active anti-cancer agents and DNA topo II inhibitors Anticancer Drug Des 1993 8 333 347 8251041

[8] Ding Q Chichak K Lown JW Pyrroloquinoline and pyridoacridine alkaloids from marine sources Curr. Med. Chem 1999 6 1 27 9873113

[9] Shinkre BA Raisch KP Fan L Velu SE Analogs of the marine alkaloid makaluvamines: synthesis, topoisomerase II inhibition, and anticancer activity Bioorg. Med. Chem. Lett 2007 10 2890 2893 17368022 10.1016/j.bmcl.2007.02.065 PMC2706148

[10] Shinkre BA Raisch KP Fan L Velu SE Synthesis and antiproliferative activity of benzyl and phenethyl analogs of makaluvamines Bioorg. Med. Chem 2008 5 2541 2549 18093835 10.1016/j.bmc.2007.11.051

[11] Wang F Ezell SJ Zhang Y Wang W Rayburn ER Nadkarni DH Murugesan S Velu SE Zhang R FBA-TPQ, a novel marine-derived compound as experimental therapy for prostate cancer Invest. New Drugs 2010 28 234 241 19274441 10.1007/s10637-009-9232-x PMC6690195

[12] Nadkarni DH Wang F Wang W Rayburn ER Ezell SJ Murugesan S Velu SE Zhang R Synthesis and *in vitro* anti-lung cancer activity of novel 1,3,4,8-tetrahydropyrrolo [4,3,2-de]quinolin-8(1H)-one alkaloid analogs Med. Chem 2009 3 227 236 19442212 10.2174/157340609788185873 PMC6690190

[13] Wang W Rayburn ER Velu SE Chen D Nadkarni DH Murugesan S Chen D Zhang R A novel synthetic iminoquinone, BA-TPQ, as an anti-breast cancer agent: *in vitro* and *in vivo* activity and mechanisms of action Breast Cancer Res. Treat 2010 10.1007/s10549- 009-0638-0 PMC3769174 19936915

[14] Geldof AA Mastbergen SC Henrar RE Faircloth GT Cytotoxicity and neurocytotoxicity of new marine anticancer agents evaluated using *in vitro* assays Cancer Chemother. Pharmacol 1999 4 312 318 10447579 10.1007/s002800050983

[15] Ryan DP Supko JG Eder JP Seiden MV Demetri G Lynch TJ Fischman AJ Davis J Jimeno J Clark JW Phase I and pharmacokinetic study of ecteinascidin 743 administered as a 72-hour continuous intravenous infusion in patients with solid malignancies Clin. Cancer Res 2001 2 231 242 11234874

[16] Na M Ding Y Wang B Tekwani BL Schinazi RF Franzblau S Kelly M Stone R Li XC Ferreira D Hamann MT Anti-infective discorhabdins from a deep-water alaskan sponge of the genus Latrunculia J. Nat. Prod 2010 26 383 387 20337497 10.1021/np900281r PMC4883701

[17] Casapullo A Cutignano A Bruno I Bifulco G Debitus C Gomez-Paloma L Riccio R Makaluvamine P, a new cytotoxic pyrroloiminoquinone from Zyzzya cf. fuliginosa J. Nat. Prod 2001 64 1354 1356 11678667 10.1021/np010053+

[18] Puchalski TA Ryan DP Garcia-Carbonero R Demetri GD Butkiewicz L Harmon D Seiden MV Maki RG Lopez-Lazaro L Jimeno J Guzman C Supko JG Pharmacokinetics of ecteinascidin 743 administered as a 24-h continuous intravenous infusion to adult patients with soft tissue sarcomas: associations with clinical characteristics, pathophysiological variables and toxicity Cancer Chemother. Pharmacol 2002 50 309 319 12357306 10.1007/s00280-002-0498-3

[19] Forouzesh B Hidalgo M Chu Q Mita A Mita M Schwartz G Jimeno J Gomez J Alfaro V Lebedinsky C Zintl P Rowinsky EK Phase I and pharmacokinetic study of trabectedin as a 1- or 3-hour infusion weekly in patients with advanced solid malignancies Clin. Cancer Res 2009 15 3591 3599 19417019 10.1158/1078-0432.CCR-08-2889

[20] Miller MJ Lonardo EC Greer RD Bevan C Edwards DA Smith JH Freeman JJ Variable responses of species and strains to white mineral oils and paraffin waxes Regul. Toxicol. Pharmacol 1996 23 55 68 8628921 10.1006/rtph.1996.0009

[21] Diwan BA Rice JM Ward JM Strain-dependent effects of phenobarbital on liver tumor promotion in inbred mice Prog. Clin. Biol. Res 1990 331 69 83 2179966

[22] Boerrigter ME Wei JY Vijg J Induction and repair of benzo[a]pyrene-DNA adducts in C57BL/6 and BALB/c mice: association with aging and longevity Mech. Ageing Dev 1995 82 31 50 7475355 10.1016/0047-6374(95)01603-w

[23] Pelleitier M Montplaisir S The nude mouse: a model of deficient T-cell function Methods Achiev. Exp. Pathol 1975 7 149 166 1105061

[24] Giovanella BC Stehlin JS Jr Williams LJ Jr Lee SS Shepard RC Heterotransplantation of human cancers into nude mice: a model system for human cancer chemotherapy Cancer 1978 42 2269 2281 719607 10.1002/1097-0142(197811)42:5<2269::aid-cncr2820420527>3.0.co;2-f

[25] Prakash C Vaz ADN Xie W Drug Metabolism: Significance and Challenges Nuclear Receptors in Drug Metabolism John Wiley & Sons Hoboken, NJ, USA 2009 1 42

[26] Agrawal S Zhang X Cai Q Zhao H Tan W Yu D Zhang R Effect of aspirin on pharmacokinetics of antisense oligonucleotides in rats J. Drug Target 1998 5 303 312 9713979 10.3109/10611869808995883

